# nSARS-CoV-2 and COVID-19 Pandemic: From Emergence to Vaccination

**DOI:** 10.1007/s44229-022-00006-x

**Published:** 2022-05-09

**Authors:** Imran Shahid

**Affiliations:** Department of Pharmacology and Toxicology, Faculty of Medicine, Umm Al-Qura University, Al-Abidiyah, P.O. Box 13578, Makkah, 21955 Saudi Arabia

**Keywords:** nSARS-CoV-2, COVID-19 pandemic, mRNA vaccines, Variants of concern, Vaccine efficacy, Vaccine breakthrough infection, Vaccine surveillance

## Abstract

Since its first emergence in Wuhan, China, the novel severe acute respiratory syndrome coronavirus-2 (nSARS-CoV-2)-associated coronavirus disease 2019 (COVID-19) has alarmingly disrupted the world’s healthcare systems and evolved as a major public health threat around the globe. Despite the advent and emergency use listing (EUL) of mRNA- and adenovirus-based vaccines to prevent the further transmission of SARS-CoV-2 infection, the pandemic burden is still significant worldwide as new cases are being reported daily. It is the first time in vaccine history that vaccines against SARS-CoV-2 have been rapidly designed, developed, and clinically evaluated and surprisingly, they have worked better than clinical trial data predicted. However, this EUL of vaccines prior to full approvals stems from the perception of inadequate testing and experience with benefit–risk balance. Similarly, the emergence of superspreader SARS-CoV-2 mutant virus strains at the end of 2020 has also raised concerns about the efficacies of approved vaccines in real-world clinical scenarios. The inconclusive, murky, and anecdotal reports about vaccine hesitancy, antibody-dependent enhancement of disease risk in vaccine injectors, and certain severe adverse events have also frightened a large segment of the world’s population, preventing them from receiving the vaccine. This review presents an overview of the remarkable efforts rendered by different vaccine producers to combat the pandemic, explains the challenges of vaccine safety and efficacies against SARS-CoV-2 variants of concern, and explores their potential roles in eradicating the COVID-19 pandemic.

## Introduction

At the time of writing this manuscript, the coronavirus disease 2019 (COVID-19) pandemic has affected 514 million people and caused 6.2 million deaths worldwide according to data released by the World Health Organization (WHO) [1]. The pandemic has also spread around the globe at a rate that has overwhelmingly disrupted many governments, halted healthcare systems, and temporarily discontinued essential healthcare services in many parts of the world. The pandemic is still ongoing and it is uncertain how long it will last. The causative agent of the pandemic, a novel beta coronavirus (i.e., severe acute respiratory syndrome coronavirus-2 [SARS-CoV-2]) was initially found to be less pathogenic with pneumonia-like illness in infected individuals at first emergence in Wuhan, China in December 2019 [2]. At that time, the mystery disease was referred to as novel CoV-2019 (nCoV-2019) and then named COVID-19 [2]. The virus transmission was faster from the epicenter of the outbreak (i.e., Hubei province, China) due to inefficient infection control measures, poor virological surveillance, and a lack of effective treatment. In the first quarter of 2020, more than 100 countries had reported cases of COVID-19 [3]. The picture became more grim when the WHO declared the global COVID-19 outbreak a pandemic on 11 March 2020 [1]. Then the epicenter of the outbreak shifted from China to Europe, Africa, and America. In the meantime, fast-spreading SARS-CoV-2 variants were identified within the virus genome sequences of infected patients from England, Africa, and Brazil, which were responsible for the rapid spread of the virus and made it more contagious [4]. Consequently, for the first time in the history of any pandemic, the world saw COVID-19 spread to every nation of the globe within the next 6 months of 2020 (from the second to third quarter of 2020) with higher morbidity and mortality rates than previous pandemics [5].

The initial treatment was symptomatic with certain repositioning drugs (e.g., azithromycin, chloroquine, hydroxychloroquine, dexamethasone), and emergency use listing (EUL) of the first drug (i.e., remedesvir; a virus polymerase inhibitor designed, developed, and tested for Ebola virus infection) in October 2020 was of great help in saving the lives of infected people at a critical stage or those in intensive care units [6]. However, those were only used as supportive therapy in asymptomatic individuals and in patients with mild to moderate disease to reverse certain signs and symptoms of the infection and slow down the viral pathogenicity [7]. In parallel, scientists and researchers worldwide, with the help and cooperation of international health organizations and the collaboration of pharmaceutical industries, started to design and develop novel vaccine candidates against SARS-CoV-2 [8]. A race soon started among the world’s leading pharmaceutical industries to develop potential candidate anti-SARS-CoV-2 vaccines while pushing drug regulatory agencies including the US Food and Drug Administration (FDA) and European Medicines Agency (EMA) to shorten traditional vaccine development and approval timeframes with steps to allow manufacturers to conduct animal studies in parallel with phase I clinical trials [2, 9]. In addition, modalities were also suggested to outline criteria for the emergency use authorization (EUA) of vaccines prior to full approval [10]. The FDA approved two vaccines (Pfizer-BioNTech and Moderna) in December 2020 and one (Janssen) in February 2021 for EUL around the world to curb the SARS-CoV-2 pandemic [2, 11]. The Oxford–AstraZeneca COVID-19 vaccine (also known as AZD1222, ChAdOx1nCoV-19) was also approved in December 2020 in the UK for EUL followed by the granting of conditional marketing authorization by the European Commission on 29 January 2021 [12]. Currently, 185 vaccines are in the development process among which 114 vaccines are in clinical evaluation, and most of them are in phase II/III clinical trials [2, 8].

It was also the first time in history of vaccinology that effective vaccines against any pandemic were designed, developed, and clinically tested within 10 months [13]. The claimed efficacies of these vaccines ranged from 70 to 95% in clinical trials against the native virus strains and surprisingly worked better in real-world administration [8, 9, 14]. Similar to their rapid development, the administration of approved anti-COVID-19 vaccines has also continued with unprecedented speed [2, 15, 16]. The recent data estimate that about 45% of the world’s population has been administered at least one dose of a COVID-19 vaccine. Similarly, 6.2 billion vaccine doses have been distributed worldwide, and 26.02 million doses are injected on a daily basis [17]. However, only 2.3% of individuals from low-income countries have received their first vaccine dose [17]. Furthermore, skepticism about vaccine safety in general, the emergence of unusual severe adverse events (SAEs) after vaccine injection, and viral breakthroughs are still hot topics of debate in the current COVID-19 vaccine administration era [15, 18, 19]. Studies have demonstrated the reemergence of measles in the USA and other parts of the world with vaccine hesitancy in recent years, and a literature review raised concerns about the safety of pandemic influenza vaccines in Europe due to the perceived risk of vaccines [20, 21]. The clinical implications of these concerns about COVID-19 vaccines are more relevant now than ever, where any doubts or suspicions about vaccine safety and efficacy will disrupt not only universal COVID-19 vaccination campaigns but also hamper other national vaccination programs [22]. In this scenario, vaccine surveillance and postmarket or postauthorization safety studies for COVID-19 vaccines are extensively required for a stronger benefit–risk profile [23].

This review article elaborates on the progress of current COVID-19 treatment developments in the form of vaccines to combat the pandemic. It also outlines concerns that have been raised about current vaccine administration with the emergence of unexpected reinfections and SAEs, low vaccine tolerance, and challenges regarding COVID-19 vaccines efficacies against the fast-spreading virus mutants. We also briefly highlight the critical importance of robust vaccine safety surveillance systems due to the development of rare SAEs after COVID-19 vaccination.

## nSARS-CoV Mutations and Variants of Concern

Although the molecular phylogeny confirms that the nSARS-CoV-2 genome shares many similarities with other beta coronaviruses, it is a novel virus [5, 24]. It makes a shift from reservoir or amplifier hosts either from animals to humans with a few unique mutations during the replication process in which it uses its replication enzymes to produce multiple copies of their genomes [23]. With the poor fidelity of the virus replication enzyme of nSARS-CoV-2 similar to other + ssRNA viruses and the lack of a mismatch repair mechanism, mutations are generated within the viral genome during the replication process [23]. These mutations generate a variant of the original virus and while improving their ability for infection, enter humans with enhanced pathogenicity and are known as variants of concern (VOCs) [25, 26]. Generally, VOCs are responsible for rapid transmission of the virus and enhanced viral infectivity, and are involved in virus immune system evasion strategies as well as the development of resistance to antiviral therapies or making the virus less susceptible to anti-COVID-19 vaccines [27]. It is also assumed that the emergence and fast spread of SARS-CoV VOC may portend a new phase/wave of the pandemic [5].

### What is the SARS-CoV-2 VOC and Variant of Interest?

According to the Centers for Disease Control and Prevention (CDC), a SARS-CoV-2 variant is a virus with a genetic code that may contain one or more mutations [3]. In the case of SARS-CoV-2, a group of variants with similar genetic changes (e.g., lineage or group of lineages) were designated by the WHO as a VOC or variant of interest (VOI) due to the shared attributes and characteristics that may require public health action. A virus lineage is a group of closely related viruses with a common ancestor. SARS-CoV-2 has many lineages, all of which cause COVID-19, and many genetic lineages have been emerging and circulating around the world since the emergence of the COVID-19 pandemic. SARS-CoV-2 VOC represents a variant for which there is evidence of increased transmissibility, more severe disease (e.g., increased hospitalizations or mortalities), a significant reduction in neutralization by antibodies generated during previous infection or vaccination, reduced effectiveness of treatments or vaccines, or diagnostic failures [3, 15]. Furthermore, declaration of a variant as a VOC might require one or more appropriate public health actions such as notification to the WHO under international health regulations, reporting to the CDC, local or regional efforts to control spread, increased screening, or research to determine the effectiveness of vaccines and treatments against the variant. In addition, based on the characteristics of the variant, additional considerations may include the development of new diagnostics or the modifications of vaccines or treatment. Since the emergence of the COVID-19 pandemic in 2019, the WHO has declared six SARS-CoV-2 variants as VOCs, including omicron (SARS-CoV-2 lineage; B.1.1.529), which is the most recent one and was first identified in South Africa with potentially increased transmissibility, a significant reduction in neutralization by some EUL monoclonal antibody treatments, and potential reduction in neutralization by postvaccination sera (Table 1). In parallel to VOC, a variant of high consequence (VOHC) has clear evidence that prevention measures or medical countermeasures have significantly reduced effectiveness relative to the previously circulating SARS-CoV-2 variant [3]. Fortunately, no SARS-CoV-2 variants have been designated a VOHC to date. Other possible attributes of a VOHC in addition to VOC include failure of SARS-CoV-2 diagnostic test targets, evidence to suggest a significant reduction in vaccine effectiveness, a disproportionately high number of infections in vaccinated persons, or very low vaccine-induced protection against severe COVID-19 disease. Similarly, increased hospitalization with more severe clinical disease and significantly reduced susceptibility to approved therapeutics or EUL are also attributed to a VOHC [3].

**Table 1:** SARS-CoV-2 variants with key mutations and their particular functions [3]

Variant	Key mutations	Lineage	Mutations function	First detected
Alpha (VOC 29–12–2020)	D614G	B.1.1.7 B.1.1.177 B.1.258	Makes the virus more infectious	UK
	delH69V70		Alters the shape of the spike (S) protein and enables the virus to evade some antibodies	
	delY144		Alters the shape of the S protein and enables the virus to evade some antibodies	
	N439K		Impacts the ability of the virus to evade antibody-mediated immunity	
	N501Y		Helps to latch the virus more tightly on human cells	
	P681H		Enhances systemic infection and increases virus transmissibility	
Beta (VOC 29–12–2020)	D512G	B.1.351 B.1.1.33	Helps to latch the virus more tightly	South Africa
	D614G		Makes the virus more infectious	
	E484K		Reduces antibody recognition by the virus and confers vaccine resistance	
	K417N		Binds the virus more tightly to human cells	
	L18F		Helps to latch the virus more tightly	
	N501Y		Helps to latch the virus more tightly on human cells	
Gamma (VOC 29–12–2020)	D614G	P.1	Makes the virus more infectious	Brazil and in Japan (detected in travelers from Brazil)
	E484K		Reduces antibody recognition by the virus and confers vaccine resistance	
	K417T		Helps to latch the virus more tightly	
	L18F		Helps to latch the virus more tightly	
	N501Y		Helps to latch the virus more tightly on human cells	
	T20N		Helps to latch the virus more tightly	
Delta (VOC 11–05–2021) Kappa (VOI 07–05–2021)	E484Q*	B.1.617.2 B.1.617.1 B.1.617.3	Similar to E484K in function while associated with immune escape and increased virus transmissibility	India
	L452R		An advantage to spread over other variants	
	P681R		Helps to latch the virus more tightly and increases virus transmissibility	
Epsilon (VOC 19–03–2021) (VOI 26–02–2021) (VOI 29–06–2021)	L452R	B.1.427 B.1.429	Reduces virus recognition to antibodies	California, USA
	S13I		Associated with variants of increased transmissibility	
Zeta (VOI 26–02–2021)	V1176F	P.2	Reduces antibody neutralization by some antibody treatments	Brazil
Eta (VOI 26–02–2021)	D614G	B.1.525 B.1.1.207	Makes the virus more infectious	UK/Nigeria
	delH69V70		Alters the shape of the S protein and enables the virus to evade some antibodies	
	E484K		Reduces antibody recognition by the virus and confers vaccine resistance	
	F888L		Reduces the antibody neutralization by some antibody treatments	
	P681H		Enhances virus systemic infection and increases virus transmissibility	
Iota (VOI 26–02–2021)	S477N	B.1.526	Reduces the susceptibility of the virus to the immune system	New York, USA
Mink variant	D614G	B.1.1.298	Makes the virus more infectious	-
Lambda (VOI 15–06–2021)	RSYLTPGD246-253 N	C.37	Resistance to viral-induced immune responses	Peru
	L452Q		Resistance to viral-induced immune responses	
	F490S		Resistance to viral-induced immune responses	
	T76I		Enhances the rate of virus transmissibility	
	L452Q		Enhances the rate of virus transmissibility	
Mu	T95I	B.1.621	Confers vaccine resistance	Colombia
	Y144S		Confers vaccine resistance	
	Y145N		Confers vaccine resistance	
	R346K		Confers vaccine resistance	
	E484K		Reduces antibody recognition by the virus and confers vaccine resistance	
	N501Y		Helps to latch the virus more tightly on human cells	
	D614G		Makes the virus more infectious	
	P681H		Enhances virus systemic infection and increases virus transmissibility	
	D109N		Confers vaccine resistance	
Omicron (VOC 26–11–2021)	A67V	B.1.1.529	Potential increased transmissibility	South Africa
	K417N		Potential reduction in neutralization by some EUA monoclonal antibody treatments	
	D614G		Makes the virus more infectious	
	E484A		Potential reduction in neutralization by postvaccination sera	

SARS-CoV-2 VOI attributes a variant with specific genetic markers that have been associated with changes to receptor binding, reduced neutralization by antibodies generated against previous infection or vaccination, reduced efficacy of treatments, potential diagnostic impact, or predicted increase in transmissibility or disease severity. Since the emergence of the COVID-19 pandemic, the WHO previously declared six SARS-CoV-2 variants to be VOIs; however, there are currently no variants designated as VOI (Table 1). Similarly, the CDC continuously monitors all SARS-CoV-2 variants worldwide and variants designated variants being monitored (VBM) include those where data indicate there is a potential or clear impact on approved or authorized medical countermeasures, have been associated with more severe disease or increased transmission but are no longer detected, or are circulating at very low levels. Furthermore, these variants do not pose a significant and immense risk to public health [3]. It is not possible to discuss here in detail SARS-CoV-2 lineages and their relevant mutations; however, Table 1 shows the classification of SARS-CoV-2 variants and their important characteristics in terms of key mutations, their functions, and prevalence.

## Impacts of VOCs on SARS-CoV-2 Biological Functionality

Scientific studies have predicted that the VOCs of SARS-CoV-2 with mutations may alter virus transmission, viral infectivity, and virulence. Some of these mutations are actively involved in altering virus binding ability to its receptors (i.e., angiotensin-converting enzyme 2 [ACE2] on the host cells) [5]. This has been most evident with the variants identified in the UK, South Africa, and Brazil, which contain these mutations and have enhanced binding to the entry receptors [27].

### Spike Protein of the SARS-CoV-2 Virus: A Hub of Key Mutations

Most of these mutations exist in the spike (S) protein of these VOCs [8, 9]. The virus S protein with a mutation at position 501 (i.e., N501Y) increases the affinity of the virus to the receptor-binding domain (RBD) of ACE2 [28] (Table 1). This mutation also enhances virulence in a mouse model. Other evidence has demonstrated higher affinities between the RBD of the S protein and host cell ACE receptors as well as increased viral transmissibility due to this site mutation [29]. In addition, the identification of a functional polybasic (Furin) cleavage site of the S protein of SARS-CoV-2 also markedly increases the binding of S protein to the ACE2 receptor, while cleaving the S protein by furin proteases [30]. The SARS-CoV-2 variant B.1.1.7 contains a mutation near the protease cleavage site (i.e., E484K) and ultimately threatens S protein stability (Table 1) [31].

### Coincidence Mutations in the SARS-CoV-2 Lineage Impact Virus Binding to Host Cells

Several variant lineages including B.1.351, B.1.1.28.1, B.1.525, and B.1.526 contain a coincidence mutation (i.e., E484K) that is located at a very critical position in the receptor-binding motif (RBM) of the RBD [5, 32]. The RBM is considered the core motif of the RBD of the S protein and is relatively less conserved and structurally highly variable; however, it directly impacts binding to the host cellular ACE2 receptors while interacting with the core functional residues of human ACE2 [32]. It has been postulated that this mutation may enhance the resistance of the SARS-CoV-2 variants to neutralize several human serum monoclonal antibodies [33]. Furthermore, this mutation can also significantly prevent virus recognition by human serum polyclonal antibodies [5, 33]. Recent studies have also predicted that this mutation is involved in changing the antigenicity of SARS-CoV-2 [34]. Hence, it could be a predisposing reason that the SARS-CoV-2 novel variant B.1.351, which harbors this mutation, is able to successfully escape the host immune system [35]. Another mutation, K417, was found to have minimal impact on the binding ability of SARS-CoV-2 to the human ACE2 receptor [5, 36]. However, both K417N/T and N501Y mutations were found to be 100% fatal in aged male mice [37]. This finding might provide a clue as to why variant 501Y.V2 is very virulent and lethal in the aged population [5, 37].

### Mutations in the Open Reading Frames of SARS-CoV-2 Impact Viral Replication Fitness

In addition to S protein mutations, some vital mutations in the open reading frames (ORFs) of nSARS-CoV-2 were also found to impact viral replication fitness [5]. The SARS-CoV-2 variant Δ382 in Singapore with a deletion of 382 base pairs in ORF8 is associated with higher replicative fitness in vitro; however, individuals affected with the variant do not show significant differences in viral load compared with the wild-type SARS-CoV-2 strain [38]. Interestingly, SARS-CoV-2 variants with ORF8 deletion mutations in some studies reflect that the inactivation of ORFs of SARS-CoV-2 might involve the adaptive evolution of SARS-CoV-2 [38]. Other deletion mutations have also been identified in multiple SARS-CoV-2 lineages, which play an essential role in the viral escape of host immunological responses [39]. One SARS CoV-2 variant (i.e., N439K) containing HV 69–70 deletion was found to be partially involved in evasion of the human immune system, whereas another Y453F found in mink potentially enhance virus binding affinity to the host cellular ACE2 receptors [40]. The data on genetic drift in SARS-CoV-2 are still not sufficient to draw any conclusion; however, due to the continuous accumulation of mutations within the virus genome, the virus may acquire immunological resistance or other altered molecular characteristics with the current worldwide spread of SARS-CoV-2 VOC [5]. It has also been shown that these mutations by chance express similar substitutions at the mutation sites in those variants [41]. Although SARS-CoV-2 variants have been reported in geographically distinct locations, the emergence of variants with coincidently identical substitutions within the mutation sites may indicate that the evolution and emergence of mutations within SARS-CoV-2 VOC share some similarities [5, 24]. It was first reported by Pfizer and BioNtech in February 2021 that mutations N501Y and Y453F expressed in two subspecies of SARS-CoV-2 of the UK and South African variants impact their effects on vaccine efficacy in phase I clinical trials [42], although phase II trials are ongoing for confirmation [42]. Hence, the characterization of these mutations would be pivotal for evaluating the efficacies of current vaccines against SARS-CoV-2 VOC, preventing virus transmission, and identifying novel treatments against the COVID-19 pandemic [5, 42].

## The Current Landscape of Candidate COVID-19 Vaccines

It is also quite interesting in the history of vaccinology that potential candidate vaccines against the COVID-19 pandemic were designed, developed, and approved for EUL worldwide by the FDA and WHO within 10 months after the first full nucleotide sequence of SARS-CoV-2 prototype strain was released by Chinese health authorities. During that period, unprecedented efforts were applied to deploy many different vaccine platforms. This race is continuing; about 185 potential candidates vaccines are in preclinical development and 114 have entered clinical development [3] (Tables 2, 3).

**Table 2:** Potential SARS-CoV-2 vaccine candidates in preclinical/clinical development [17]: different SARS-CoV-2 vaccine platforms in preclinical and clinical development

Platform		Candidate vaccines	
		*n*	%
PS	Protein subunit	39	34
VVnr	Viral vector (non-replicating)	17	15
DNA	DNA	11	10
IV	Inactivated virus	16	14
RNA	RNA	19	17
VVr	Viral vector (replicating)	2	2
VLP	Virus-like particle	5	4
VVr + APC	VVr + antigen-presenting cell	2	2
LAV	Live attenuated virus	2	2
VVnr + APC	VVnr + antigen-presenting cell	1	1
		114

**Table 3:** Different SARS-CoV-2 vaccine platforms in terms of the number of doses and schedule

Number of doses and schedule		Candidate vaccines	
		*n*	%
One dose		16	14%
Day 0		16	
Two doses		74	65%
Day 0 + 14		6	
Day 0 + 21		28	
Day 0 + 28		40	
Three doses		1	1%
Day 0 + 28 + 56		1	
To be defined/no data		23	20%
Total		114	
Route of administration			
Oral		3	3%
Injectable		96	84%
SC	Subcutaneous	5	4%
ID	Intradermal	4	4%
IM	Intramuscular	87	76%
IN	Intranasal	8	7%
To be defined/no data		15	13%

In addition to considering traditional vaccine formulation methods based on inactivated, live or attenuated-virus, protein subunits, or virus-like particles, first-time novel and promising mRNA vaccines that deliver information to synthesize the SARS-CoV-2 antigen protein (i.e., S- protein) have also been exploited [8]. Conventional vaccine approaches based on adenoviral vectors and DNA platforms are very effective, safe, have been ubiquitously used for many years and are still in use [8]. However, the norms of mRNA vaccine usage in humans are still unknown and not extensively studied in humans [8]. It is the first time that mRNA-based vaccines (i.e., mRNA-1273, BNT162b1/ BNT162b2) were developed against SARS-CoV-2 and are the first-in-class licensure for EUL worldwide in adults over age 18, approved by the FDA on 11 and 18 December 2020, respectively [43–47]. The EUL for the BNT162b1 and BNT162b2 COVID-19 vaccines to include adolescents 12 through 15 years of age was expanded on May 10, 2021. The FDA issued an EUL for the third vaccine Ad26.COV2.S on 27 February 2021 [48]. To date, 13 anti-COVID-19 vaccines have either been approved by the FDA or WHO for EUL or have clinical efficacy and safety data from phase III clinical trials [3]. Table 4 lists all vaccines approved or EUL of the FDA, CDC, or EMA that have been administered worldwide to curb the COVID-19 pandemic.

**Table 4:** Vaccines developed against COVID-19 with their efficacy data, doses, and eligibility criteria from phase III clinical trials

Clinical trial regime	Vaccine manufacturer and name	Vaccine platform	SARS-CoV-2 lineage at the time of trial	Endpoint measures to determine the vaccine efficacy	Overall vaccine efficacy, results by severity, and eligibility
Two doses (21 days apart)	Pfizer–BioNTech (BnT162b2)	mRNA	B.1.351, P.1, B.1.427/B.1.419, P.2, B.1.526	Symptomatic COVID-19 and positive RT-PCR test result	95%, 100% effective in preventing CDC-defined severe disease; 95.3% effective in preventing FDA-defined severe disease > 16 years old
Two doses (28 days apart)	Moderna (mRNA-1273)	mRNA	B.1.427/B.1.429, B.1.526	Symptomatic COVID-19 and positive RT-PCR test result	94%, 100% efficacy against severe disease, ≥ 18 years old (12 years old to younger than 18 years (NCT04649151) and 6 months old to younger than 12 years (NCT04796896))
Two doses (< 6 weeks apart), two doses (> 12 weeks apart)	AstraZeneca– University of Oxford (AZD1222 (Vaxzevria, also called Covishield when manufactured by SII under license))	Viral vector	B.1.1.7, B.1.351, P.1, B.1.427/B.1.429, P.2, B.1.526, C.37	Symptomatic COVID-19 and positive NAAT result	55–81%, 100% efficacy against hospitalization, ≥ 18 years old (WHO); ≥ 40 years old and not pregnant in the UK
One dose	Johnson & Johnson (Ad26.CoV2-S)	Viral vector	B.1.351, P.1, B.1.427/B.1.429, P.2, B.1.526, C.37	Symptomatic COVID-19 and positive RT-PCR test result	66%, 85.4% efficacy against severe–critical disease occurring ≥ 28 days after vaccination, ≥ 18 years old
Two doses (21 days apart)	Gamaleyab (Sputnik V)	Viral vector	No variants have been identified originating from the trial locations from the trial start date to the present (June 2021)	Symptomatic COVID-19 and positive RT-PCR test result	92%, No data available (June 2021), ≥ 18 years old
Two doses (28 days apart)	Bharat Biotech (Covaxin)	Viral vector	Phase III trial began on 16 November 2020 and is ongoing in India; variants identified include B.1.617.2 and B.1.617.1	Symptomatic COVID-19 and positive RT-PCR test result at least 14 days after the second dose	78%, 100% efficacy against hospitalization, ≥ 18 years old (2–18 years old: study ongoing)
Two doses (14 days apart; 14 or 28 days apart in Chile)	Sinovac Biotech (CoronaVac)	Inactivated virus	P.1 and P.2	Symptomatic, virologically confirmed COVID-19 occurring from 2 weeks after the second dose up to 1 year after the first dose	Multiple studies in different countries: 50.7% (Brazil), 56.5% (Chile), 65% (Indonesia), 78% (Brazil) and 91% (Turkey), 51% efficacy against symptomatic SARS-CoV-2 infection; 100% efficacy against severe disease; 100% efficacy against hospitalization from 14 days after second dose, ≥ 18 years old
Two doses (21 days apart)	Sinopharm (BBIBP-CorV)	Inactivated virus	No variants have been identified originating from the trial locations during this time (June 2021)	Occurrence of COVID-19	78%, 79% efficacy against hospitalization, ≥ 18 years old
Two doses (21 days apart)	Novavax (NVX-CoV2373)	Protein subunit	B.1.1.7, B.1.351, B.1.427/B.1.429, B.1.526	Symptomatic COVID-19 and positive RT-PCR test result at least 7 days after the second dose	89%, 100% efficacy against severe disease and hospitalization, ≥ 18 years old (12–17 years old: study ongoing, NCT04611802)
Two doses (21–28 days apart)	VECTOR (EpiVacCorona) (NCT04780035)	Protein subunit	No variants have been identified originating in the trial locations during this time (June 2021)	Symptomatic COVID-19, laboratory-confirmed COVID-19 within 6 months after the first dose	No data available (June 2021), No data available (June 2021), ≥ 18 years old

*CDC* Centers for Disease Control and Prevention; *FDA* US Food and Drug Administration; *NAAT* nucleic acid amplification test; *RT-PCR* reverse transcription-polymerase chain reaction; *SARS-CoV-2* severe acute respiratory syndrome coronavirus 2

### Current Scenario of Worldwide COVID-19 Vaccine Application

**Fig. 1:**
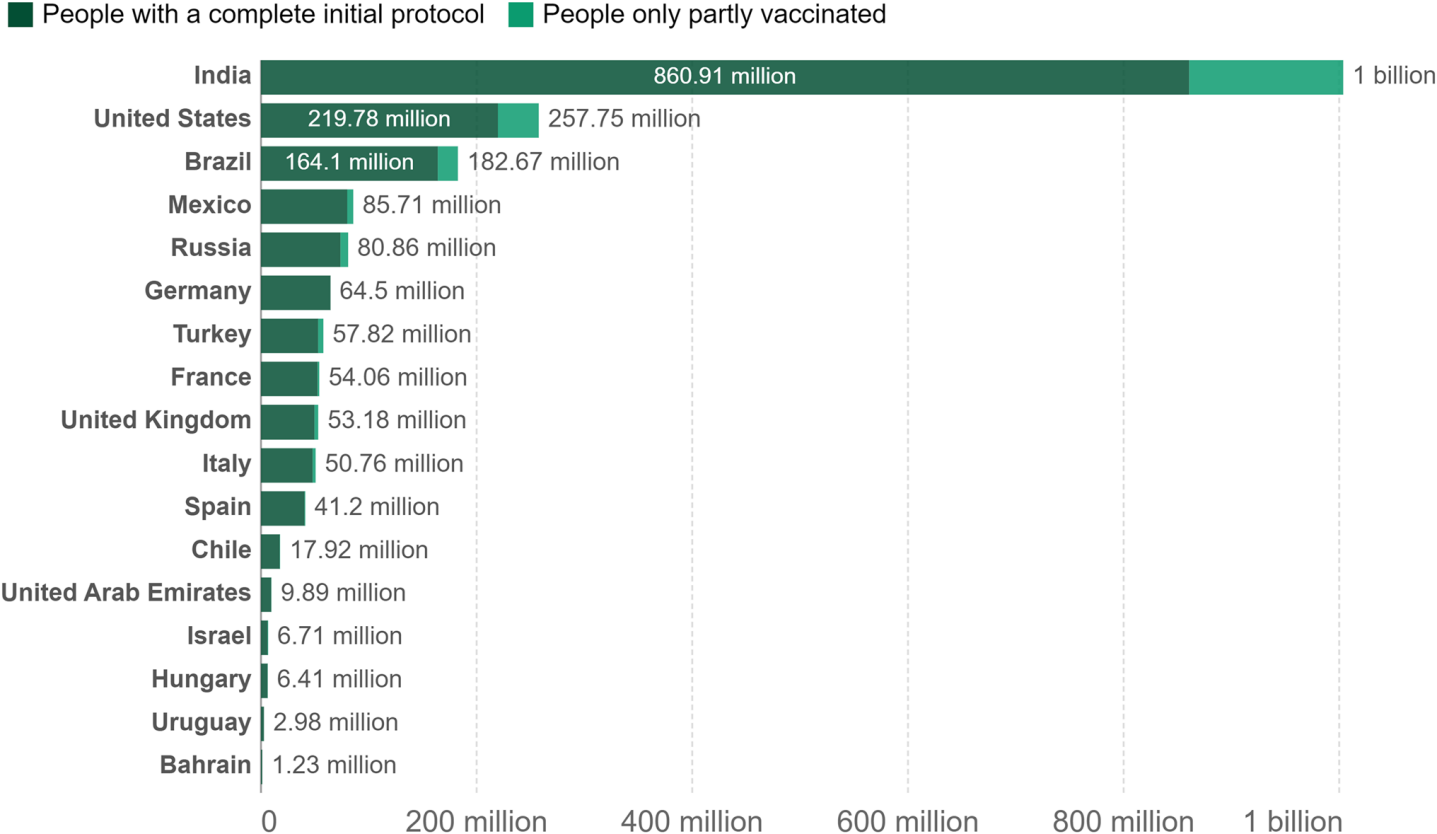
Number of people vaccinated against COVID-19: Alternative definitions of full vaccination, (e.g., having been infected with SARS-CoV-2 and having one dose of a two-dose protocol) were ignored to maximize comparability between countries. These data are only available for countries that reported the breakdown of doses administered by the first and second doses in absolute numbers [17]

**Fig. 2:**
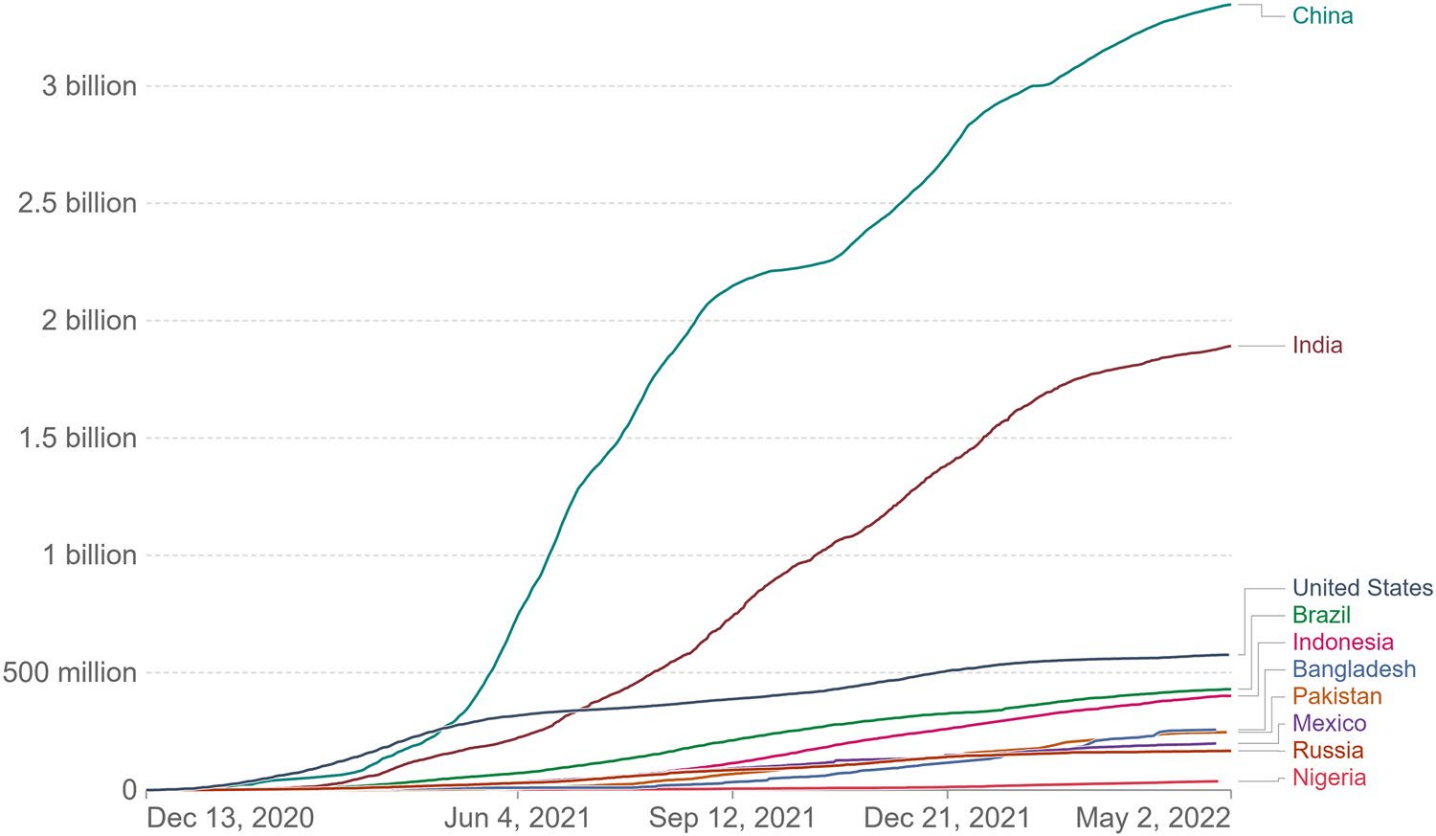
COVID-19 vaccine doses administered: For vaccines that require multiple doses, each dose is counted. As the same person may receive more than one dose, the number of doses can be higher than the number of people in the population [17]

Until now, the worldwide application of COVID-19 vaccines has seemed effective, well tolerable, and safe (Figs. 1, 2); however, side effects and some very rare SAEs have been documented worldwide in a fraction of the vaccinated population [5, 43–50]. According to CDC and WHO, the ratio of non-SAEs was reported in about 372 cases per million doses of mRNA vaccines in the USA [1, 3, 14, 17]. Surprisingly, this ratio was higher for the adenoviral vector-based vaccine ChAdOx1 (AZD1222) in the UK, where about 4000 AEs per million doses of this vaccine were recorded by the UK safety monitoring system [1, 3, 14, 17]. The ongoing phase I/II clinical trial data of an inactivated virus vaccine (e.g., CoronaVac) and two vaccines already in the pipeline developed by Sinopharm have demonstrated that the side effects associated with these vaccines are manageable and not serious [1, 3, 14, 17]. To date, no deaths have been directly attributed to administration of the COVID-19 vaccines [1, 3]. Safety concerns regarding currently used COVID-19 vaccines are not only due to the reported side effects or because of rare SAEs but also due to the emergence of SARS-CoV-2 VOCs [5, 27].

As aforementioned, currently developed and deployed COVID-19 vaccines are based on the genome of the wild-type SARS-CoV-2 strain (i.e., prototype strain); the clinical efficacy and safety of these vaccines against VOCs is still unclear and compromised in real-world clinical scenarios [27]. More clinical data are required to determine the clinical effectiveness of these vaccines in SARS-CoV-2 VOC-affected populations as well as to investigate even very rare AEs following the vaccination of large populations [49–51].

Unfortunately, lessons learned from the development of vaccines against previous SARS-CoV and Middle East respiratory syndrome coronavirus (MERS-CoV) infections were limited regarding safety and effectiveness in humans because none had moved forward to regulatory approval, distribution, and application in humans due to successful public health containment and healthcare measures to control and prevent SARS-CoV- and MERS-CoV-related disease outbreaks in the recent past [5, 8, 9]. In this section, we briefly overview the safety and efficacy of COVID-19 vaccines against SARS-CoV-2 wild and variant strains, challenges, and predictive barriers for COVID-19 vaccine postmarket studies to recognize potential risks or AEs of special interest while administering the vaccines to large populations.

### Safety and Efficacy of mRNA COVID-19 Vaccines

#### mRNA-1273 Vaccine

Clinical trials to assess the safety and efficacy of the mRNA-1273 vaccine were conducted at 99 centers across the USA (COVE trial; COVE ClinicalTrials.gov number NCT04470427) in which the volunteers (*n* = 30,420) were assigned in a 1:1 ratio to receive either vaccine mRNA-1273 (100 μg) or placebo 28 days apart [46]. Overall vaccine efficacy was 94.1% [95% confidence interval (CI) 89.3–96.8] with confirmed symptomatic COVID-19 infection in 185 participants in the placebo group and 11 in the vaccine group [46]. The vaccine efficacy was 95.6% for participants aged 18 to < 65 years and 86.4% for those > 65 years [46]. SAEs were rare and mostly pertained to local and systemic reactions that were found with similar frequency in both groups, and no safety concerns were demonstrated. The mRNA-1273 vaccine elicits immune responses to protect the recipient (i.e., binding antibody responses, neutralizing antibody titers, and type 1 helper T-cell responses) approximately 10 days after the first dose; however, maximum protection is reached after the second dose [46]. The median 2-month follow-up of the Moderna vaccine (mRNA-1273) showed no safety concerns in the clinical trial, and the frequency of unsolicited AEs and rare SAEs during or after two injections was similar in both the vaccinated and placebo groups. However, the frequency of solicited AEs at the injection site was relatively higher (i.e., 88.6%) in vaccine recipients than placebo recipients after administration of the second dose [46]. The safety, immunogenicity, and reactogenicity studies of the Moderna vaccine in elderly individuals are very important, as this fraction of the COVID-19-affected population has an increased rate of severe illness and death [46]. In contrast to adults < 65 years of age, solicited AEs were predominantly mild or moderate in severity with the Moderna vaccine in adults > 65 including fatigue, chills, headache, myalgia, and pain at the injection site [46]. Neutralizing antibody responses and strong CD4 cytokine responses with the involvement of type 1 helper T cells were elicited and documented to be similar in vaccine recipients between the ages of 18 and 55 years [46]. Interestingly, higher neutralizing-antibody titers were reported in vaccine recipients receiving the 100 µg dose than the 25 µg dose, which supports the administration of 100 µg mRNA-1273 vaccine in phase III clinical trials to adults > 65 years [46].

#### BNT162b1/BNT162b2 mRNA Vaccine

The BioNTech and Pfizer vaccine BNT162b2/BNT162b1 was the first mRNA-based vaccine to receive FDA EUL in persons > 16 years with a two-dose regimen (30 μg per dose) to confer protection against COVID-19 [44, 45]. Overall vaccine efficacy was 95% (95% CI 90.3–97.6) with two doses of BNT162b2 21 days apart in the vaccine group (*n* = 21,720) than the placebo group (*n* = 21,720) in phase III clinical trials (Table 5) [45]. Phase II/III part of global phase I/II/III clinical trial data with reported safety and efficacy were the basis for the EUL application of the vaccine [45]. Only 8 cases of COVID-19 with onset at least 7 days after receiving the second vaccine dose were reported among the volunteers, and 162 cases were reported among placebo recipients [45]. The vaccine efficacy was between 90 and 100% across various patient subgroups based on several demographic characteristics, clinical triage spectrum, and comorbidities (e.g., blood pressure, diabetes) (Table 5) [45]. The vaccine side effects were quite manageable and included injection-site pain, myalgia, and headache [45]. There was a low incidence of SAEs in both the vaccine and placebo groups. Vaccine safety was similar to that of other viral vaccines over a median of 2 months [45].

**Table 5:** Comparative analyses of vaccine effectiveness against different outcomes of dominant SARS-CoV-2 alpha VOCs [15, 16]

Outcomes	Vaccine effectiveness			
	Pfizer-BioNTech	Oxford-AstraZeneca		
	One dose	Two doses	One dose	Two doses
Symptomatic COVID-19 infections/disease	55–70%^a^	85–95%^a^	55–70%^a^	70–85%^b^
Hospitalization of COVID-19 patients	75–85%^a^	90–99%^b^	75–85%^a^	80–99%^c^
Mortality	70–85%	95–99%^b^	75–85%^b^	75–99%^c^
COVID-19 infection	55–70%^b^	70–90%^c^	55–70%^b^	65–90%^c^
COVID-19 transmission (secondary cases)	45–50%^c^	No data	35–50%^c^	No data

^a^High confidence. Evidence from multiple studies which is consistent and comprehensive

^b^Medium confidence. Evidence is emerging from a limited number of studies or with a moderate level of uncertainty

^c^Low confidence. Little evidence is available at present and results are inconclusive

### Human Adenovirus Vector Vaccine (Ad26.COV2.S)

The third vaccine Ad26.COV2.S, which received FDA EUL in February 2021, was designed by the Janssen Biotech company and is a recombinant, replication-competent human adenovirus type 26 vector encoding full-length SARS-CoV-2 S protein in a prefusion-stabilized conformation [48]. The vaccine efficacy was determined in an international, randomized, double-blind, placebo-controlled, phase III clinical trial (ENSEMBLE ClinicalTrials.gov number NCT04505722) [48]. Adult SARS-CoV-2-negative participants (*n* = 19,630) were administered a single dose of the vaccine, and 19,691 volunteers received a placebo [48]. The vaccine efficacy against moderate to severe–critical COVID-19-affected participants was 66.9% (95% CI 59.0–73.4) with onset at least 14 days after administration, and 66.1% (95% CI 55.0–74.8) at least 28 days after administration [48]. However; for severe–critical COVID-19 cases, vaccine efficacy was significantly higher at 76.7% (95% CI 54.6–89.1) with onset at ≥ 14 days, and 85.4% (95% CI 54.2–96.9) with onset at ≥ 28 days [48]. Interestingly, vaccine reactogenicity was higher compared with placebo; nevertheless, it was mild to moderate and transient [48]. The frequency and incidence of SAEs were almost similar and balanced between the two groups [48]. Overall, a single dose of this vaccine was found to be protective against symptomatic/asymptomatic SARS-CoV-2 infection and effective against severe–critical COVID-19 patients in the hospital. The safety profile of the vaccine was almost similar to other COVID-19 vaccines in phase III clinical trials [48].

### NVX-CoV2373 Vaccine

Recent clinical data on the NVX-CoV2373 vaccine (Novavax) showed that the vaccine is safe and elicits robust immune responses in healthy adult individuals [49]. Phase III clinical trials demonstrated that a two-dose vaccine regimen in adult individuals conferred 89.7% protection against COVID-19 infection and showed high efficacy against the SARS-CoV-2 VOC B.1.1.7 [49]. Phase III randomized, observer-blinded, and placebo-controlled clinical trials were conducted at 33 sites in the UK. The adult participants (*n* = 15,187) between the ages of 18 and 84 years were divided at a 1:1 ratio into vaccine recipient and placebo groups; 27.9% of the participants were > 65 years, and 44.6% had coexisting morbidities [49]. The vaccine group received two intramuscular 5-μg doses of the vaccine or placebo administered 21 days apart [49]. Overall vaccine efficacy was 89.7% (95% CI 80.2–94.6) with the onset of infection symptom reported in 10 vaccinated participants and 96 in the placebo group at least 7 days after administration of the second dose [49]. No hospitalization or casualties were documented among the 10 COVID-19 symptomatic cases in the vaccinated individuals [49]. Vaccine reactogenicity was mild and transient, and the ratio of SAEs incidences was low and similar in both groups [49]. Post hoc analyses showed the vaccine efficacy was 86.3% (95% CI 71.3–93.5) against the SARS-CoV-2 VoC B.1.1.7 (alpha) and 96.4% (95% CI 73.8–99.5) against non-B.1.17 variants [49].

### Side Effects and AEs of COVID-19 Vaccines

Although all FDA-approved or EUL COVID-19 vaccines have undergone or continue to undergo the most intensive safety monitoring by using both established and new safety monitoring systems to make sure that COVID-19 vaccines are safe; however, vaccine-induced side effects and rare AEs have been reported worldwide [3]. The side effects that happened within 7 days of getting the vaccine are common but mostly mild to moderate. Some individuals may experience reactions that affect their ability to do daily activities. After the second dose of the vaccine, side effects may experience throughout the body such as fever, tiredness, and headache. Local or systemic minor acute reactions (e.g., injection pain, swelling and redness, joint and muscle pain) are also commonly reported after the COVID-19 vaccination [52]. A recent study documented increased reactogenicity of heterologous prime-boost regimens compared with homologous vaccination with the administration of a combination of AZD1222 and BNT162b2 vaccines. The rollout of COVID-19 vaccines across multiple countries, targeting millions of recipients has also led to more SAEs including anaphylaxis, myocarditis, and thrombocytopenia [52]. As of February 2021, 66 cases of anaphylaxis have been documented among 17,524,676 mRNA vaccines recipients in the USA [52]. It was presumed that this could be related to the polyethylene glycol-based components of the mRNA vaccines. Most cases were reported in females (i.e., 92%; 63 of 66) who were administered an adrenaline injection as part of the emergency treatment. No casualties have been reported to date of anaphylaxis following COVID-19 vaccination. Several cases of heart inflammation (myocarditis and pericarditis) were reported through the CDC Vaccine Adverse Event Reporting System after vaccination with both mRNA vaccines (i.e., either mRNA-1273 or BNT162b2) in May 2021. As of July 2021, 5166 cases of myocarditis have been reported for the BNT162b2 vaccine, and 399 cases have been documented for the mRNA-1273 vaccine among 129 million vaccinated individuals in the USA. Myocarditis has also been reported in individuals vaccinated with mRNA-1273 in Israel [52]. However, the reports are rare about myocarditis and pericarditis in adolescents and young adults who have received one of the two doses of mRNA COVID-19 vaccines, and these cases occur more often after getting the second dose. The potential benefits of the mRNA1273 vaccine outweigh the known and potential risks, including myocarditis or pericarditis [3, 15].

The EMA concluded a causal link between AZD1222 administration, blood clotting, and low platelet counts (i.e., thrombocytopenia) in vaccinated individuals leading to 30 deaths in March 2021 [52]. Subsequently, the vaccine agency in the European countries and the UK issued age-based restrictions on the administration of AZD1222. Similarly, rare thrombocytopenia events were documented with some casualties (six deaths of more than 6.8 million vaccinated individuals) following the administration of Ad26.COV2-S vaccine in the USA because of this, the FDA briefly paused the use of Ad26.COV2-S in April 2021. First, it was hypothesized that adenovirus-based vaccines are strongly associated with thrombocytopenia; however, it was also observed after mRNA vaccine administration. The rate of vaccine-induced immune thrombotic thrombocytopenia occurrence was found to be different in different regions, with a higher rate reported in Scandinavia (1 in 10,000) than in the UK which reflects regional differences among human leukocyte antigens, vaccine sensitivity, and preexisting conditions [52].

## Clinical Impacts of the SARS-CoV-2 VOC on COVID-19 Vaccine Safety and Efficacy

Apart from novel potential candidate vaccines in the development phase, the vaccines’ effectiveness against the emerging SARS-CoV-2 variants has become a hotspot issue of global discussion. How SARS-CoV-2 variants clinically impact the efficacy and safety of COVID-19 vaccines are still not clearly understood, and data from clinical trials and real-life vaccinations are limited for conclusive evidence (Table 5). However, recent data reported from different studies around the world project that the current vaccines are proportionally less effective against the SARS-CoV-2 variant lineages or variants decrease the efficacy and safety of the current vaccines compared with non-variant infections (i.e., wild-type SARS-CoV-2 strains) [31, 49–51, 53].

### Impacts of SARS-CoV-2 VOCs on COVID-19 mRNA Vaccine Efficacy

Previous studies and recent reports of some COVID-19 reinfection in fully vaccinated individuals have demonstrated that these variants may significantly impact the efficacy and safety of COVID-19 vaccines [54–57]. A study by Shi et al. evaluated the neutralization of BNT162b2 vaccine-elicited sera by using engineered mutant SARS-CoV-2 viruses. For this purpose, three SARS-CoV-2 mutant variants were constructed by the investigators including the N501Y, 69/70-deletion-N501Y-D614G, and E484K-N501Y-D614G variants [58, 59]. All of these mutant variants exhibited minimal effects on the neutralization of 20 vaccine-elicited sera. Another study by Wang et al. [59] evaluated the impacts of SARS-CoV-2 variants on the neutralization of antibodies and memory B-cell responses in 20 mRNA-1273 or BNT162b2 vaccine recipients. The study’s findings revealed that the neutralizing activity of vaccine-elicited sera was reduced against the tested SARS-CoV-2 pseudoviruses containing E484K, N501Y, and K417N-E484K-N501Y mutations.

### Impacts of SARS-CoV-2 VOCs on the Efficacy of Other COVID-19 Vaccines

SARS-CoV variants with E484K mutation have been shown to significantly reduce the neutralizing activity of human convalescent and postvaccination sera [60]. In one study, the neutralization phenotype of pseudoviruses containing 501Y.V1, 501Y.V2, and P.1 mutants was assessed against convalescent sera, the vaccine-elicited sera of mRNA-1273 and NVX-CoV2373, and monoclonal antibodies [60]. The study’s findings demonstrated decreased neutralizing activity; however, one potential limitation of this study was that the engineered pseudoviruses were not presenting the biological characteristics of the prototype SARS-CoV-2 strain [60]. By contrast, one study conducted in February 2021, reported that the neutralizing and protective activity of two vaccines (BBIBP-CorV and ZF2001) was still significant against the variant lineage 501Y.V2, although the neutralization titer of postvaccination sera declined 1.6-fold [61]. The findings of the study also indicated that the virus variant 501Y.V2 exhibited more resistance to vaccine-elicited sera [61]. A study by Cele et al. [62] also reported similar findings, showing that the convalescent plasma of patients infected with no-CoV variant (i.e., the variant usually expressing the D614G mutation) exhibited reduced neutralizing ability to the 501Y.V2 SARS-CoV-2 variant. However, the convalescent plasma from patients infected with the 501Y.V2 variant demonstrated a moderate reduction in neutralizing activity to the no-CoV variant. A study by Wang et al*.* [63] described the immunological resistance of the SARS-CoV-2 variants to antibody neutralization by using convalescent sera and sera from the recipients of inactivated-virus vaccines (BBIBP-CorV or CoronaVac). The findings revealed that the neutralization activity of convalescent sera or vaccinated elicited sera against SARS-CoV-2 variant B.1.1.7 was slightly reduced but was significantly reduced against the B.1.351 variant [63]. Both variants also induced higher resistance against the CoronaVac-elicited serum than the prototype SARS-CoV-2 [63]. Apart from these studies, several other in vitro experiments and clinical trials are underway to investigate the immunological resistance of SARS-CoV-2 variants to neutralizing antibodies or T-cell immune responses [63].

Recent phase III clinical trial data of NVX-CoV2373 on vaccine efficacy demonstrated that the vaccine has different efficacies against SARS-CoV2 variants 501Y.V1 (B.1.1.7) and 501Y.V2 (B.1.351) [58]. The protective efficacy of the vaccine is > 85% against the variant 501Y.V1 and < 50% against 501Y.V2 [58]. The findings also showed that these virus variants challenge the efficacy and safety of recombinant protein subunit vaccines as well as the mRNA and human and chimpanzee adenoviral vector-based vaccines as previously mentioned [58].

The available clinical data also indicate that the virus genome variants confer resistance to vaccine-induced immunity [5, 27]. Detailed studies are needed on the variants’ kinetics to host cell immune response adaptations, virus immune evasion strategies, and infection propagation to design better vaccine models. There is also an urgent need for alternate treatment strategies in the form of direct-acting antivirals with a high genetic barrier to resistance and the production of specific monoclonal or polyclonal antibodies against the challenging SARS-CoV-2 variants.

## COVID-19 mRNA Vaccines for Adolescents and Children

### Efficacy and Safety of COVID-19 mRNA Vaccines in Adolescents

On 10 May 10 2021, the FDA authorized the Pfizer-BioNTech COVID-19 vaccine for emergency use in adolescents 12 through 15 years of age as a significant step in the fight against the COVID-19 pandemic. From 1 March 2020 through 30 April 2021, approximately 1.5 million COVID-19 cases in individuals 11–17 years of age have been reported to the CDC [15]. Children and adolescents generally have a milder COVID-19 disease course compared with adults. The Pfizer-BioNTech COVID-19 vaccine is administered as a series of two doses, 3 weeks apart, at the same dosage and dosing regimen for ≥ 16 years of age. The FDA determined that the Pfizer-BioNTech COVID-19 vaccine met the statutory criteria to amend the EUA, and that the known and potential benefits of this vaccine in individuals ≥ 12 years of age outweigh the known and potential risks, supporting the vaccine’s use in this population. Two recent papers reported the safety, immunogenicity, and efficacy of the Pfizer-BioNTech COVID-19 vaccine in adolescents [64, 65]. In one study conducted in 2260 adolescents 12–15 years of age, BNT162b2 had a favorable safety and side-effect profile, with mainly transient mild to moderate reactogenicity (i.e., predominately injection-site pain; 79%), fatigue (60%), and headache (55%). Except for a few overall SAEs, no serious vaccine-related AEs were reported. The observed vaccine efficacy was 100% and no COVID-19 cases with an onset of 7 or more days after the second dose were noted among BNT162b2 recipients without evidence of previous SARS-CoV-2 infection compared with 16 cases that occurred among placebo recipients [64]. The second ongoing phase II/III placebo-controlled trial reported an acceptable safety profile of the mRNA-1273 vaccine in adolescents. The immune response of the vaccine was similar to that in young adults and the vaccine was found to be efficacious in preventing COVID-19. The most common solicited AEs after the first or second vaccine injections were injection-site pain (93.1% and 92.4% respectively), headache (44.6% and 70.2% respectively), and fatigue (47.9% and 67.8% respectively). No vaccine-related SAEs were noted. Furthermore, no cases of COVID-19 with an onset of 14 days after the second dose of mRNA vaccine were reported compared with four cases that occurred in the placebo group [65].

### COVID-19 mRNA Vaccine Trials in School-Aged Children

As the COVID-19 pandemic continues, infants and school-aged children remain at risk of severe disease requiring hospitalization, multisystem inflammatory syndrome, and even death. Vaccinating younger children may provide hope that the significant impact of the COVID-19 pandemic on all children could be diminished in the future [66]. An increased proportion of COVID-19 cases in children (under 18 years of age) was reported in the USA, where about 700 children have died since the pandemic emerged. COVID-19 vaccines received EUL for children older than 12 years in May 2021; however, safe and effective vaccines against COVID-19 are urgently needed in children younger than 12 years of age. A phase I dose-finding study and an ongoing phase II/III randomized trial are being conducted to investigate the safety, immunogenicity, and efficacy of the two doses of BNT162b2 vaccine administered 21 days apart in children 6 months to 11 years of age [66]. The study also presented the vaccine efficacy results for children aged 5–11 years. The study findings revealed that two 10-µg doses of BNT162b2 (Pfizer-BioNTech) vaccine 21 days apart were safe and effective in children aged 5–11 years. Most AEs were local (i.e., pain at the injection site), transient, and most often reported following the 30-µg dose instead of the 10-µg vaccine dose. Overall vaccine efficacy was reportedly 90.7%, and COVID-19 with onset 7 days or more after the second dose was reported in three recipients of the BNT162b2 vaccine compared with 16 placebo recipients [66].

## Real-World Challenges to SARS-CoV-2 Vaccines

### COVID-19 Vaccine Breakthrough and COVID-19 Reinfections in Vaccine Jabs

The current anti-SARS-CoV-2 vaccines are facing several challenges regarding their administration, distribution, efficacies, and safety in real-life situations despite their significant contributions in decreasing the number of COVID-19-associated comorbidities and mortalities as well as preventing the transmission of pandemic worldwide. However, some studies have reported asymptomatic or mild symptomatic COVID-19 reinfections in individuals fully vaccinated for longer than 2 weeks, also known as “COVID-19 vaccine breakthrough” cases [54–57]. This specific time point (i.e., 2-week mark) is essential as it is when the human body may develop immunity. It raises concerns about vaccine efficacies and safety in the elderly in whom the immune system is already compromised due to other ailments or in elderly individuals who recently recovered from the COVID-19 infection. Furthermore, mRNA-1273 or BNT162b2 vaccines are claimed to be 95% effective in preventing serious COVID-19 illnesses in clinical trials, which means that out of 100 vaccinated individuals, 5 are not able to elicit higher antibody-neutralizing responses to provide protection [43–47]. Consequently, the safety profile of these vaccines remains unclear and minor illness may occur. It was also expected during the clinical trials of these vaccines that some breakthrough cases may occur, but the investigators were primarily focused on whether the participants develop symptoms [54–57]. In a study where 14 cases of vaccine breakthrough infections were identified in health care providers (HCPs), illnesses were reportedly mild or with no symptoms although they tested positive during routine screening [57]. It is not unusual for both of these vaccines to allow some breakthrough cases, as the same experiences have occurred in clinical trials of already used vaccines (e.g., flu vaccines) [54]. The occurrence of vaccine breakthroughs provides a reminder to follow precautions even after getting the vaccine. People still need to wear a mask, maintain social distancing, and follow guidelines as adopted during the peak pandemic era. HCPs in hospitals, clinics, and health care centers must report any cases where COVID-19 testing is underway and any positive case at least 14 days after completing their second vaccine dose [31, 54–57].

### COVID-19 Vaccine-Associated SAEs

Although SARS-CoV-2 vaccine-induced SAEs are very rare, the tested efficacy and safety of the current vaccines in the small number of participants during clinical trials could not lead decisive conclusions to be drawn about their actual severity in vaccinated persons [11, 31, 54–57]. Furthermore, the time limit to report, access, and evaluate those SAEs were very short during the clinical trials for the practical reasons of completing the trial as soon as possible and applying for EUL by the vaccine manufacturers [94]. In many cases, the researchers relied on trial volunteers to report symptoms of AEs and SAEs and present them for testing [94]. A common factor among all of the vaccine recipients was reactogenicity, which might have diverted their attention from considering minor AEs due to COVID-19 vaccines rather than injection-site reactogenicity and therefore made them less likely to refer recipients for testing [67]. Another important aspect relates to the rate of asymptomatic COVID-19 infection during vaccination, which has not yet been reported during the safety and efficacy trials of these vaccines [67].

### Dosage Algorithm and Logistic Concerns for COVID-19 Vaccines

Other important concerns also require additional studies on the safety and clinical efficacy of these vaccines. First, when the number of administered vaccine doses increases to millions and possibly billions, unexpected safety issues will arise [67], which leads to the question of how the emerging SAEs will be managed with or without longer follow-ups. Second, the implementation of a vaccine with two doses also seems challenging if a large proportion of the population denies or misses a second vaccine dose [67]. Third, it remains unknown how long the COVID-19 vaccines will remain effective. Fourth, the data are scarce regarding the effectiveness of the vaccines against asymptomatic COVID-19 infection and limiting transmission. Fifth, to eradicate the pandemic, the kinetics and dynamics of neutralizing antibodies and protective immune responses to the virus are still uncertain and unknown [67]. However, phase I follow-up studies of the mRNA-1273 vaccine demonstrated the persistent activity of neutralizing antibodies until 3 months after the second dose of vaccine. Finally, an important consideration is the important fractions of vaccine recipient populations (e.g., children, pregnant women, and immunocompromised patients of various sorts) who were not included in the clinical trials and in whom vaccine efficacy and safety data are limited [67]. From manufacturing and logistic aspects, the preparation, transportation, and delivery of the vaccines at − 70 °C are also challenging and daunting in some settings with resource constraints as well as their deployment in low-to-middle-income countries [67].

### COVID-19 Breakthrough Infections with SARS-CoV-2 VOC

Despite the high efficacies of currently administered vaccines, COVID-19 breakthrough infections and vaccine breakthrough infections with SARS-CoV-2 variants have been rarely reported in vaccinated HCPs [57]. However, data in this regard are needed to extensively elucidate the correlation between breakthrough and infectivity. From the clinical data of 1497 fully vaccinated HCPs in the largest medical center in Israel, 39 SARS-CoV-2 breakthrough infections were documented [57]. Neutralizing antibody titers among breakthrough infected HCPs during the preinfection period were lower compared with uninfected controls (case-to-control ratio, 0.361); 95% CI 0.165–0.787) [57]. Seventy-four percent of breakthrough infection cases were found to have a high viral titer at some point during their infection, although 59% (*n* = 17) had a positive concurrent antigen-detecting rapid diagnostic test [57]. Most breakthrough infections were reportedly mild or asymptomatic; however, 19% of patients had persistent symptoms for more than 6 weeks, and 85% of the tested samples were found positive for the B.1.1.7(alpha) SARS VOC [57].

In another study of a cohort of 417 individuals administered a second dose of BNT162b2 or mRNA-1273 vaccine at least 14 days prior, two females reported vaccine breakthrough variant infections [56]. Interestingly, in both women, COVID-19 infection signs and symptoms developed despite the strong evidence of vaccine efficacies and positive screening by PCR test [56]. The viral genome sequencing revealed VOCs of likely clinical importance, with E484K mutations in one female and three other mutations (i.e., T951, del142-144, and D614G) in both females [56]. These findings reveal that a significant risk of COVID-19 breakthrough infection with variant virus strains still exists even after successful vaccination and strongly emphasizes genomic surveillance studies to detect and characterize SARS-CoV-2 VOCs [56].

## Limitations of the Study

This is the first time in the history of virology that investigators, researchers, and scientists around the world have made dedicated efforts to vanquish a viral pandemic by designing and developing state-of-the-art diagnostic tools and novel vaccine platforms to prevent the further transmission of the virus and treat infections. The current manual of SARS-CoV-2 diagnostics and COVID-19 treatment research is expanding and quite dense as interesting findings and pragmatic scientific ideas are published daily to eliminate the pandemic. Hence, it is not possible to cover every aspect of this pandemic starting from SARS-CoV-2 epidemiology to treatment in this review. Instead, we mainly focused on updates about virus circulating genotypes and lineages, along with their clinical impact on virus transmission and infectivity, and finally the role of current vaccines in eradicating this pandemic worldwide.

## Conclusions

At the time of writing this review, a single dose of COVID-19 vaccines has been administered to about 44.9% of the world’s population. Despite these tremendous efforts of continuous vaccine distribution worldwide and administration to COVID-19-affected and noninfected individuals to avert the virus transmission and to slow down the complexity and severity of COVID-19-associated comorbidities, the devastating effects of the pandemic on human health are ongoing. The vaccine-associated SAEs, continuous emergence of viral VOCs/VOIs, and the incidences of COVID-19 breakthrough infections with SARS-CoV-2 variants are adding additional hurdles to achieve universal vaccination against the pandemic as well as herd immunity to combat infection infectivity. Furthermore, the lack of vaccine for neonates, infants, and children < 12 years of age as well as the lack of availability of safe dosage algorithms for pregnant females and women of reproductive age are also raising concerns about the safety and efficacies of current COVID-19 vaccine regimens. Epidemiological and genome surveillance studies will be crucial to frame the path and trajectory of the COVID-19 pandemic as well as the completion of the universal vaccination program and the design, development, and deployment of alternate novel treatment strategies in the form of direct-acting antivirals and anti-mRNA based treatment strategies, which will be pragmatic and fruitful to end the pandemic.
